# Integrative Systems-Level Transcriptomic Network Analysis Identifies Candidate Genes Associated with Biofilm Formation and Virulence in *Pseudomonas aeruginosa*

**DOI:** 10.3390/ijms27125407

**Published:** 2026-06-16

**Authors:** Sara H. Mohamed, Asmaa Reda, Tarek A. Yousef, Mona G. Nada, Maha S. I. Wizrah, Sahar A. Mandour

**Affiliations:** 1 Department of Microbiology, Egyptian Drug Authority (EDA), Formerly National Organization for Drug Control and Research (NODCAR), Giza 12654, Egypt; sara_hussein_moh@yahoo.com; 2 Computational Biology and Bioinformatics Division, Zoology Department, Faculty of Science, Benha University, Benha 13518, Egypt; 3 Center of Nanotechnology, King Abdulaziz University, Jeddah 21589, Saudi Arabia; 4 Department of Chemistry, College of Science, Imam Mohammad Ibn Saud Islamic University (IMSIU), Riyadh 11623, Saudi Arabia; tayousef@imamu.edu.sa; 5 Department of Medical Microbiology and Immunology Department, Faculty of Medicine, Cairo University, Cairo 11956, Egypt; mona.nada@kasralainy.edu.eg; 6 Department of Biology, College of Science and Humanities in Al-Kharj, Prince Sattam Bin Abdulaziz University, Al-Kharj 11942, Saudi Arabia; m.wizrah@psau.edu.sa; 7 Department of Microbiology and Immunology, Faculty of Pharmacy, Deraya University, New Minia 61111, Egypt; sahar.mandour@deraya.edu.eg

**Keywords:** *Pseudomonas aeruginosa*, biofilm, WGCNA, pathogenesis, drug-resistant, bioinformatics

## Abstract

*Pseudomonas aeruginosa* (*P. aeruginosa*) is a multidrug-resistant opportunistic pathogen that causes both acute and chronic infections and is known for its ability to form biofilms. In the current study, we applied a hypothesis-generating framework primarily based on integrating four different datasets and applying batch correction. Weighted Gene Co-Expression Network Analysis (WGCNA) was performed in parallel with differential expression analysis using *limma*. Therefore, we aimed to identify potential biofilm-associated gene candidates. Significant candidate genes were subjected to functional analysis and gene ontology, followed by the construction of a protein–protein interaction network using STRING. The *Pseudomonas* Genome Database was used to highlight the candidate genes. A total of 271, 687, 533, and 277 significantly up-regulated differentially expressed genes (DEGs), as well as 306, 985, 472, and 312 significantly down-regulated DEGs, resulted from the exploratory analysis. Through WGCNA/limma integration, 223 common significantly up-regulated/positively correlated gene candidates were identified. Functional analysis results showed significant enrichment in virulence-related pathways, such as biofilm formation (*PA0083*, *PA0084*, *hcp1*, *hcpC*, *pilH*, *pilI*, *pilJ*, *vfr*, *pqsA*, *pqsB*, *pqsC*, *pqsE*, *PA1657*, and *PA1658*). In addition, other virulence-related pathways, such as quorum sensing, phenazine biosynthesis, the bacterial secretion system, and secondary metabolite biosynthesis, were enriched. In conclusion, our hypothesis-generating integrative analysis identifies candidate genes and potential pathways associated with biofilm formation, virulence, and other processes in *P. aeruginosa*. In light of this, we point out that all candidate genes presented in this study remain hypothesis-generating. Further validation is recommended, including large-scale in silico analyses and in vitro experimental studies.

## 1. Introduction

*Pseudomonas aeruginosa* (*P. aeruginosa*) is a multidrug-resistant opportunistic pathogen that causes acute or chronic infections [1,2]. It has recently been included in the World Health Organization’s priority bacterial pathogens list [3] as one of the high-priority pathogens deemed of greatest concern due to the requirement for new antibiotics. These pathogens make treatment challenging because they exhibit resistance to multiple commonly used antimicrobials. These options are currently considered the most effective drugs for treating multidrug-resistant (MDR) pathogens [4,5].

*P. aeruginosa* strains can develop resistance to commonly used antimicrobials. When dealing with known drug-resistant strains, MDR can be resistant to numerous drugs but can remain susceptible to one or two classes of antimicrobials. On the other hand, difficult-to-treat (DTR) strains have far fewer treatment options available [5,6]. To date, the treatment of *P. aeruginosa* infections remains very difficult due to the pathogen’s well-documented resistance to multiple antimicrobials. Also, the virulence of *P. aeruginosa* involves biofilm formation, secretion of toxins and enzymes, and production of secondary metabolites that promote immune evasion and tissue damage [7,8].

Resistance mechanisms can be acquired, adaptive, or intrinsic. Adaptive tolerance and persistence, including biofilm formation, can reduce susceptibility to commonly used antibiotics such as aminoglycosides, quinolones, and β-lactams [9,10]. Moreover, an interesting feature of *P. aeruginosa* biofilms is the assembly of a multilayered polymeric matrix. This biofilm’s structure protects the bacteria from external doses of many conventional antibiotics. Therefore, it is considered one of the major factors that impacts its clinical significance [4,11]. These biofilms may also survive in hypoxic environments and extreme conditions. Therefore, the treatment of their related infections has become challenging due to their rapid spread, tolerance, and resistance [12].

Using advanced molecular biology techniques, we can now discover and identify genes involved in bacterial pathogenesis, referred to hereafter as virulence genes [13]. Relying solely on known virulence genes can limit the accuracy of discovering and identifying pathogenicity labels. Therefore, by using comprehensive approaches, we can examine all genes present in both pathogenic and non-pathogenic bacteria [14]. In bacterial pathogenesis, hub genes are highly connected members of a network that may play important roles in infection and disease development [15]. Therefore, they are considered potentially important targets to study [13,16]. Additionally, they could help bacteria survive under stress, including low nutrient availability or antibiotic exposure [17]. Hub genes play such an important role that they are being investigated as potential new targets for antibacterial therapies [15,18]. Therefore, bacterial spread can be prevented by blocking these hubs and/or inducing the bacterial system to halt its growth, virulence, or ability to cause disease [15,18].

Therefore, our study computationally integrates independent datasets of *P. aeruginosa* biofilm producers with batch-effect correction. Here, we aimed to apply a bioinformatics framework for hypothesis generation that achieves robustness across different datasets. Although recent large-scale studies provide important information [19], integrative studies can provide additional perspectives. In our study, to reduce inter-dataset heterogeneity, we followed standardized preprocessing steps and treated the biological variability in our datasets as a conservative filtering strategy. This approach aimed to identify central candidate genes potentially associated with the biofilm growth mode of *P. aeruginosa* and to determine their roles in virulence and pathogenicity.

## 2. Results

### 2.1. Identification of Common Significantly Up- and Down-Regulated Differentially Expressed Genes (DEGs) Across All Selected Datasets (Exploratory Analysis)

**Figure 1:**
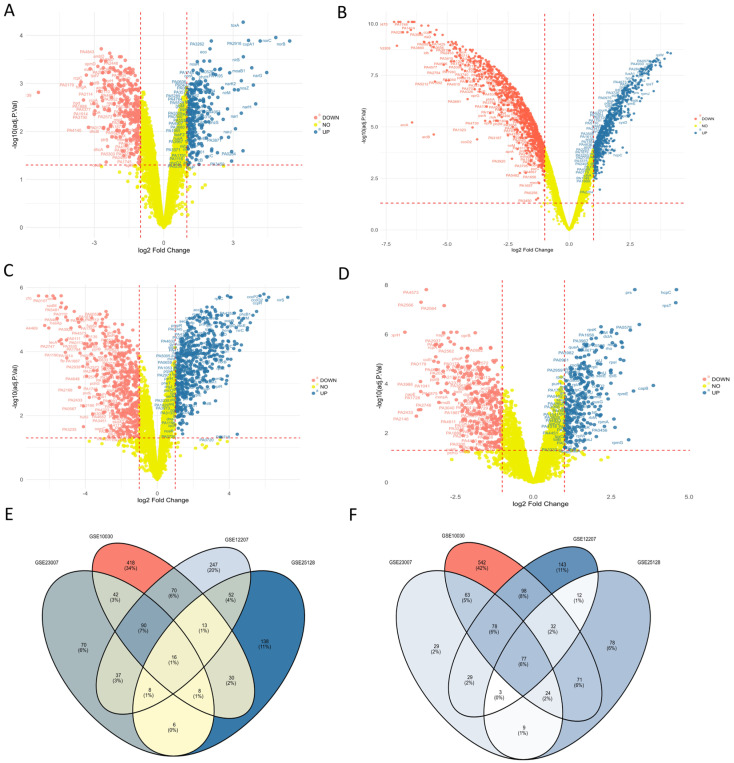
Detection of common up- and down-differentially expressed genes. (**A**–**D**) Volcano plots representing up- and down-DEGs for GSE25128, GSE10030, GSE12207, and GSE23007. (**E**,**F**) Showing a Venn diagram representing the DEGs intersection among up- and down-DEGs, respectively. Significance criteria were adjusted as adjusted *p*-value < 0.05 and |log2 fold change|  >  1.

In this work, four microarray datasets (GSE25128, GSE10030, GSE12207, and GSE23007) were pre-processed and normalized (Supplementary Materials Figures S1–S4) and analyzed using the limma package to detect significant up- and down-regulated DEGs. As a result, according to significance criteria (adjusted *p*-value < 0.05 and |log2 fold change| >  1), 271, 687, 533, and 277 significant up-DEGs were detected, respectively. Similarly, 306, 985, 472, and 312 significant down-DEGs were identified, respectively (Figure 1A–D). Using the Venn diagram, 16 common up-regulated DEGs were obtained across the four datasets. These genes include *hcpC*, *secY*, *nusA*, *argG*, *rplO*, *rpmB*, *rplL*, *PA5130* (*yibN*), *PA3822* (*yajC*), *adk*, *PA4933*, *rpsR*, *rpmD*, *infB*, *pnp*, and *rpmG*, and 77 common down-DEGs were shared (Figure 1E,F).

### 2.2. Identification of Candidate Genes Potentially Associated with Biofilm Formation by a Combined Approach (WGCNA/Limma)

**Figure 2:**
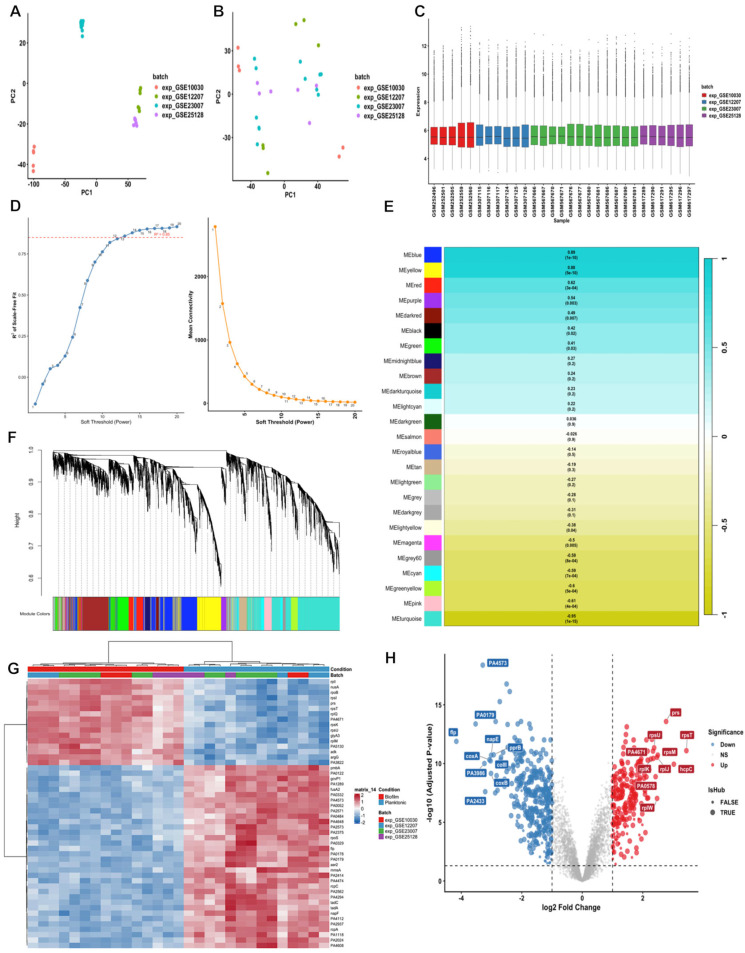
Weighted Gene Co-Expression Network Analysis (WGCNA) of *Pseudomonas aeruginosa* biofilm. (**A**,**B**) Datasets before and after batch correction. (**C**) Expression distribution of all samples. (**D**) The scale-free topology fit index (R^2^) and average connectivity are plotted versus the soft-thresholding powers. (**E**) Heatmap of module eigengene correlations showing relationships between modules. (**F**) Clustering dendrogram of genes showing co-expression modules identified by dynamic tree cutting. (**G**) Heatmap showing the DEGs identified using limma after the batch effect removal. (**H**) Volcano plot showing the up- and down-DEGs identified by limma after the batch effect removal.

The pre-processed/normalized matrices were subjected to the WGCNA pipeline. After batch correction, Figure 2A,B indicate that the technical batch effects were removed. As shown in Supplementary Materials Figure S5, sampling clustering was performed as a sample quality check step, along with the expression distribution (Figure 2C).

In our study, the same combined/batch-corrected data were analyzed using WGCNA and differential expression analysis (limma) in parallel. In the WGCNA pipeline, the soft-thresholding power was set to 13, as determined by both automatic power selection (using pickSoftThreshold), scale-free topology fit, and mean connectivity values (Figure 2D). As shown in Supplementary Materials Table S1, the selected power achieved both a scale-free topology (R^2^ > 0.85) and a high mean connectivity of 50.8.

Co-expression modules were identified using a clustering dendrogram and dynamic tree cutting. Modules with a distance of less than 0.25 were then merged using mergeCutHeight. This process resulted in 25 modules (Figure 2E,F). Seven significant modules (*p*-value < 0.05) were identified: blue, yellow, red, purple, dark red, black, and green modules. These modules showed correlations of >0.4, indicating moderate to high correlation (Supplementary Materials Table S2). These modules are accordingly associated with our trait (biofilm growth mode). A total of 2295 genes were identified across the 7 significant modules and subjected to further analysis. The clustering dendrogram showed many modules, each represented by a different color, that were detected by the dynamic tree cutting (Figure 2F). Module-trait relationships presented as a heatmap showed the correlation coefficients between the module eigengenes and the biofilm phenotype (Figure 2E). This thereby identified the modules most significantly associated with this phenotype.

The limma pipeline was applied to the combined data after the batch-effect removal step. A total of 579 significant DEGs were identified, including 224 significantly up-regulated genes related to the biofilm and 355 significantly down-regulated genes. As presented in the heatmap (Figure 2G), the genes showed perfect discrimination between the biofilm and planktonic phenotype. The volcano plot (Figure 2H) highlights the significant DEGs, which will be subjected to further analysis.

In our work, to select more significant candidate genes, we intersected the two results (positively correlated genes from WGCNA and significant up-DEGs from limma), yielding 223 common significant genes. Those were considered candidate genes and subjected to further analysis (Supplementary Materials Figure S6).

### 2.3. Pathway, Functional Enrichment Analysis, and Hub Candidate Gene Detection

**Figure 3:**
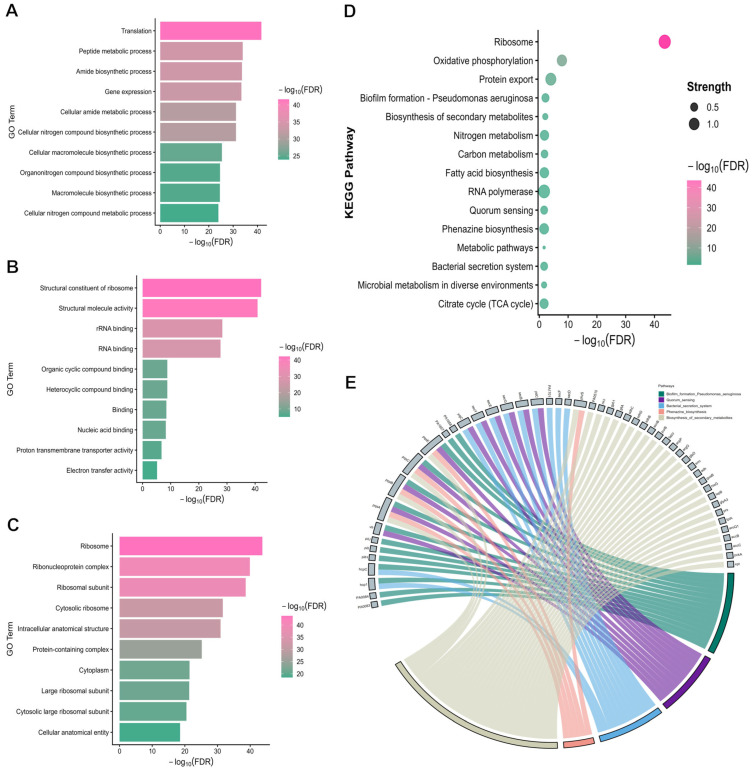
Gene ontology (GO) and functional analysis of significant candidate genes extracted from positively correlated WGCNA modules. (**A**–**C**) Bar plots showing the GO BP, MF, and CC, respectively. (**D**) Dot plot showing significant KEGG pathways. (**E**) Circus plot showing candidate genes enriched in biofilm-related KEGG pathways. Color intensity (**A**–**D**) represents statistical significance. Pathway annotation: Colors represent enriched biological pathways (**E**) as Green: biofilm formation (*Pseudomonas aeruginosa*), Purple: quorum sensing, Blue: bacterial secretion system, Orange: phenazine biosynthesis, and Beige: biosynthesis of secondary metabolites.

To go deeper, we extracted 223 significant candidate genes in common and used them to construct a PPI network. Functional analysis was performed to assess the role of these modules in the context of biofilm growth mode in *P. aeruginosa*. Regarding the gene ontology results (Figure 3A–C), the most significant GO BP enrichment was Translation (FDR = 2.30 × 10^−42^). This was followed by the terms Peptide metabolic process (FDR = 1.12 × 10^−34^) and Amide biosynthetic process (FDR = 2.40 × 10^−34^). The most significant GO MF terms were structural constituent of ribosome (FDR = 7.23 × 10^−43^), structural molecule activity (FDR = 1.35 × 10^−41^), and rRNA binding (FDR = 4.63 × 10^−29^). The top significant GO CC term was ribosome (FDR = 1.82 × 10^−44^). This was followed by the Ribonucleoprotein complex (FDR = 1.22 × 10^−40^), and ribosomal subunit (FDR = 2.30 × 10^−39^) terms. KEGG analysis identified ribosome (FDR = 2.80 × 10^−44^), oxidative phosphorylation (FDR = 1.42 × 10^−8^), and protein export (FDR = 1.00 × 10^−4^) as the most significant pathways (Figure 3D). However, some significant KEGG pathways are linked to biofilm formation and potential virulence in *P. aeruginosa* (Figure 3E). As shown in Figure 3D,E, a “Biofilm formation-*Pseudomonas aeruginosa*” pathway (FDR = 7.20 × 10^−3^) was obtained. This pathway includes *PA0083*, *PA0084*, *hcp1*, *hcpC*, *pilH*, *pilI*, *pilJ*, *vfr*, *pqsA*, *pqsB*, *pqsC*, *pqsE*, *PA1657*, and *PA1658* genes. Quorum sensing (FDR = 2.15 × 10^−2^), having *vfr*, *pqsA*, *pqsB*, *pqsC*, *pqsE*, *yajC*, *secY*, *secE*, *secG*, *secB*, and *yidC* genes. Phenazine biosynthesis (FDR = 2.15 × 10^−2^), having *pqsA*, *pqsB*, *pqsC*, *pqsE*, and *phzS* genes. Bacterial secretion system (FDR = 2.15 × 10^−2^), having *hcp1*, *hcpC*, *PA1701*, *secF*, *secD*, *yajC*, *secY*, *secE*, *secG*, *secB*, and *yidC* genes. Biosynthesis of secondary metabolites (FDR = 8.60 × 10^−3^), having *PA0510*, *nirJ*, *gph1*, *pqsA*, *pqsB*, *pqsC*, *pqsE*, *gltA*, *sdhC*, *sdhD*, *sdhB*, *acnB*, *purB*, *fabV*, *acpP*, *argG*, *glpD*, *eno*, *adk*, *guaB*, *phzS*, *hisG*, *ispB*, *glyA3*, *prs*, *tpiA*, *aroQ1*, *accB*, *accC*, *pckA*, and *xpt* genes.

**Figure 4:**
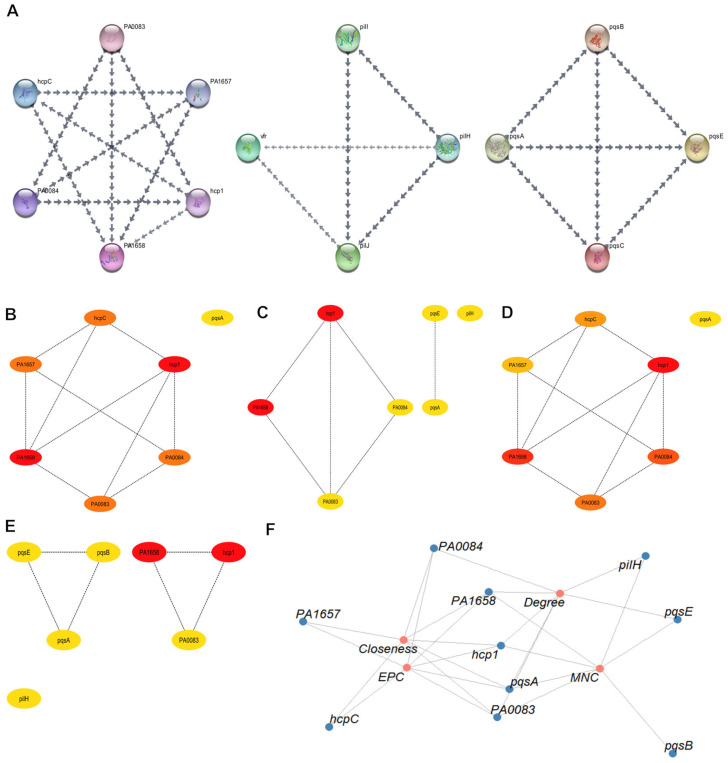
Protein–protein interaction (PPI) network, and candidate genes detection using significant candidate genes enriched in biofilm formation–*Pseudomonas aeruginosa* KEGG pathway. (**A**) A protein–protein interaction (PPI) network was constructed in STRING with a high-confidence threshold of >0.7. (**B**–**E**) Hub gene networks were identified in Cytoscape using the CytoHubba plugin with the algorithms Closeness Centrality, Degree Centrality, MNC (Maximum Neighborhood Component), and EPC (Edge Percolated Component). (**F**) Network showing the intersection of hub genes across different algorithms. Tested candidate genes: *PA0083* (*tssB1*), *PA0084* (*tssC1*), *hcp1*, *hcpC*, *pilH*, *pilI*, *pilJ*, *Vfr*, *pqsA*, *pqsB*, *pqsC*, *pqsE*, *PA1657* (*hsiB2*), and *PA1658* (*hsiC2*).

To provide an integrated overview and identify candidates for future studies, various algorithms were applied. In our study, candidate genes enriched in the “Biofilm Formation-*Pseudomonas aeruginosa*” KEGG pathway were identified. A high-confidence > 0.7 PPI network was constructed using STRING (Figure 4A). Hub gene networks were observed in Cytoscape (v 3.10.2) using the CytoHubba plugin (Figure 4B–E). Herein, we applied different algorithms of Closeness Centrality, Degree Centrality, MNC (Maximum Neighborhood Component), and EPC (Edge Percolated Component). Across all four algorithms, four genes (*pqsA*, *hcp1*, *PA0083* (*tssB1*), and *PA1658* (*hsiC2*)) were shared. These results may represent high-confidence hub genes in this biofilm network and may be included in its mechanism. Also, some candidate genes were shared between three of the four algorithms (*pqsE*, *PA0084* (*tssC1*), and *pilH*). Finally, three genes (*PA1657* (*hsiB2*), *hcpC*, and *pqsB*) were shared between only two algorithms (Figure 4F).

### 2.4. Functional, Mechanistic Characterization, and In Silico Validation of Biofilm-Associated Hub Candidate Genes

To prioritize functionally relevant candidate genes involved in *P. aeruginosa* biofilm-associated growth mode, multi-domain biological annotation was used. From the most promising candidate gene list, we identified a total of 14 candidate genes enriched in the biofilm formation KEGG pathway (Supplementary Materials Table S3).

Data presented in Supplementary Materials Table S3 showed the involvement of several critical mechanisms. This suggests their potential roles in biofilm formation in *P. aeruginosa*. *PA0083* (*tssB1*) and PA0084 (*tssC1*) are involved in the Hcp secretion island I type VI secretion system. *Hcp1* and *HcpC* are secreted structural components of the T6SS. *PA1657* (*hsiB2*) and *PA1658* (*hsiC2*) are associated with protein secretion by the type VI secretion system. *PilH*, *PilI*, and *PilJ* are genes involved in type IV pilus-mediated motility and signal transduction. The four genes (*pqsA*, *pqsB*, *pqsC*, and *pqsE*) were also detected and are known to play roles in the quorum-sensing system. The *Vfr* transcriptional regulator gene was included in the two-component system, quorum sensing, and biofilm formation according to the KEGG results. Some of our genes have been classified by KEGG as part of the ribosome pathway.

The candidate genes identified from our integration analysis were evaluated using an independent *P. aeruginosa* PAO1 dataset as a preliminary validation (Supplementary Materials Figure S7). Our results provide preliminary in silico support for a subset of the candidate genes observed across time-dependent and independent analyses. This result should be interpreted with several considerations. As a hypothesis-generating study, our analysis was primarily based on integrating four different datasets using multiple pipelines. In contrast, validation with a single external dataset of limited sample size may reduce statistical power. In addition, it may limit the reproducibility of the identified candidate genes. Furthermore, platform-related differences between studies may contribute to inconsistencies in individual gene-level validation. Therefore, analysis of the external dataset was included to provide preliminary computational evidence in support rather than definitive confirmation of all candidate genes.

## 3. Discussion

The bacterial transcriptome is a naturally occurring, dynamic entity. Changes in bacterial transcript levels are considered a key factor in the adaptive response to environmental changes and other stress conditions [20,21,22]. Data integration approaches that combine multiple datasets offer several advantages over a single-study design [23]. The novelty of our study lies in the application of an integrated hypothesis-generating pipeline to publicly available *P. aeruginosa* microarray data. Reprocessing of all datasets was performed using a standardized pipeline. Our steps include batch-effect removal followed by parallel analyses with the WGCNA and *limma* pipelines. Thereby, we aimed to emphasize signals that consistently appear across experiments and to enhance the identification of true *P. aeruginosa* mechanisms/patterns.

Our analysis identified 223 candidate genes (Supplementary Materials Figure S6). Functional analysis in the STRING database identified 15 significant KEGG pathways (Figure 3D). Several potential virulence-associated pathways linked to biofilm formation and related processes were identified (Figure 3E). QS is an intercellular communication system that can regulate both bacterial virulence and biofilm formation [24,25]. In this regard, it was discussed whether QS in *P. aeruginosa* could alter biofilm formation, suggesting that it could serve as a possible strategy [26]. Biosynthesis of secondary metabolites is another potential virulence mechanism identified in our work. These compounds are produced by *P. aeruginosa*, enabling it to survive in host environments through virulence strategies [27]. One example of these compounds is the bacterial pigments (pyocyanin and pyoverdine). These pigments are considered significant factors in virulence and persistence across different *P. aeruginosa* infections [28,29]. Regarding phenazine biosynthesis, Schiessl et al. stated that phenazines produced by bacteria provide a degree of protection to bacteria growing in biofilm structures by resisting antibiotics [30]. Also, the occurrence of these potential virulence pathways together in our analysis suggests a functional connectivity strategy. It is not just a set of independent pathways, but may instead represent a set of linked ones. These pathways, processes, and compounds, the components they produce, may be linked in different ways and, in turn, may affect each other [31,32,33,34,35,36].

Fourteen candidate genes (*PA0083*, *PA0084*, *hcp1*, *hcpC*, *pilH*, *pilI*, *pilJ*, *vfr*, *pqsA*, *pqsB*, *pqsC*, *pqsE*, *PA1657*, and *PA1658*) were enriched in the “Biofilm formation—*Pseudomonas aeruginosa*” KEGG pathway (Supplementary Materials Table S3). Some of these candidate genes are not among the commonly used biofilm-associated markers. Commonly used detectors in *P. aeruginosa* include QS-related genes (such as *lasI*, *lasR*, *rhlI*, *rhlR*) [37,38,39]. Exopolysaccharide-related genes (such as *pel*, *psl*, *algD*) [40,41,42], and motility-related genes (such as *fliC*, *pilA*) [1,33,43,44] were also included. In our study, these genes were pointed out due to their enrichment in the biofilm formation pathway. However, candidate genes enriched in other potential virulence pathways remain important and require validation.

In our study, *HcpC* was captured in both exploratory and combined/integrative analyses. Recent studies have shown that bacterial secretion system-associated genes are involved in bacterial and host–pathogen interactions [45,46,47,48]. *TssB* and *TssC* are members of the T6SS [49]. Also, *TssC1* has been reported to play an important role in the resistance of *P. aeruginosa* biofilms to antimicrobials [50]. Another set of candidate genes (*pilH*, *pilI*, and *pilJ*) resulted from our analysis. Yarrington et al. report the chemosensory role of *PilJ* [51]. *Vfr* may be involved in regulating the virulence program of *P. aeruginosa* [52]. It has also been reported that *PqsE* promotes the production of certain alkylquinolone signaling compounds and balances secondary metabolite levels [53]. Herein, the enrichment of our candidate genes in the biofilm formation pathway, together with their reported roles in recent studies, suggests their potential involvement in *P. aeruginosa* processes.

**Figure 5:**
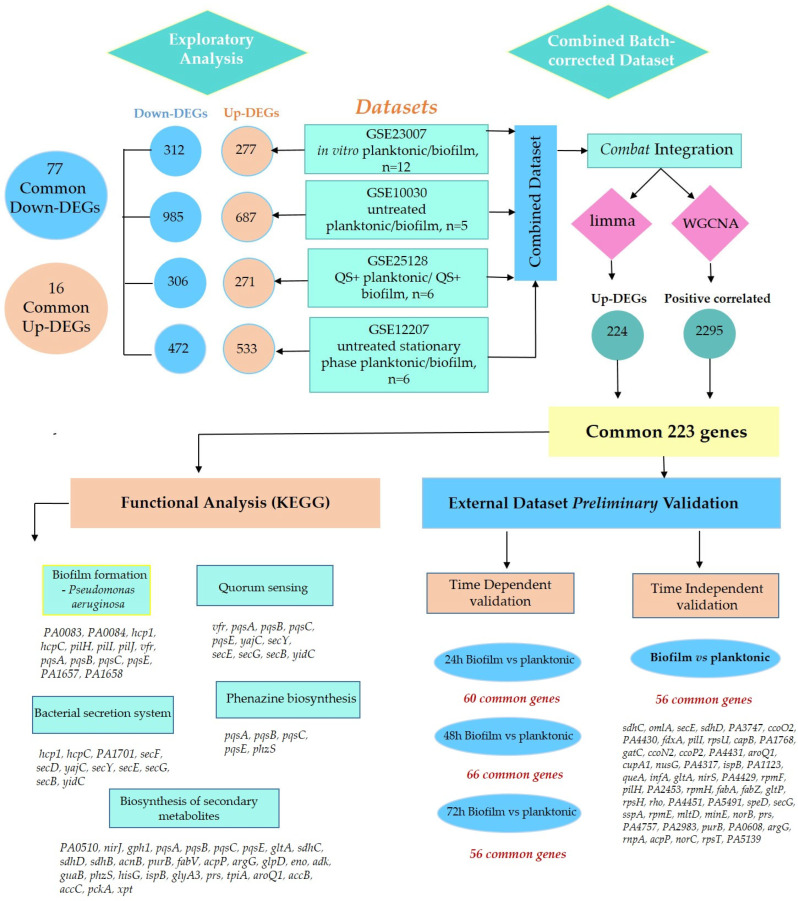
Flowchart.

In summary, our integrative analysis identified candidate genes (*n* = 223) potentially linked to *P. aeruginosa* biofilm formation, virulence, and persistence (Figure 5). A subset of these genes showed supporting evidence in an external dataset, although this validation is limited by sample size and potential platform differences. Considering this, we note that all candidate genes identified in this study remain hypothesis-generating. Further validation is recommended, including large-scale in silico analyses and in vitro experimental studies. These studies are needed to confirm their potential roles in biofilm formation, persistence, and virulence in *P. aeruginosa*.

## 4. Materials and Methods

### 4.1. Datasets Used in the Study

Four microarray raw CEL files were downloaded from the NCBI Gene Expression Omnibus (GEO, https://www.ncbi.nlm.nih.gov/geo/ (accessed on 1 October 2025)). Datasets used include GSE23007 (in vitro planktonic/biofilm, *n* = 12), GSE10030 (untreated planktonic/biofilm, *n* = 5), GSE25128 (QS+ planktonic/QS+ biofilm, *n* = 6), and GSE12207 (untreated stationary phase planktonic/biofilm, *n* = 6). Using the GEOquery (v2.72.0) package in R (version 4.4.0), both phenotypic metadata and annotations were retrieved. Herein, the criteria for selecting datasets were as follows. First, all from the same organism (*P. aeruginosa*). Second, contained data for both planktonic and biofilm growth modes. Third, all datasets were obtained from the same type of microarray platform (GPL84) to reduce experimental bias. Fourth, all datasets were publicly available in CEL format for consistent preprocessing.

#### Rationale for Dataset Heterogeneity

The four datasets vary in biofilm formation time and culture conditions (Supplementary Materials Table S4). Rather than treating biological differences across datasets as a limitation, we intentionally leveraged them as a filter to identify core biofilm-associated candidate genes. In doing so, we aim to remove genes associated with specific stages or specific conditions. On the other hand, prioritizing other genes present across all our studied experimental conditions. This is consistent with our objective to discover a significant transcriptional signature associated with *P. aeruginosa* biofilm formation. In addition, this increases confidence in the candidates that pass all filters.

### 4.2. Data Pre-Processing and Quality Control (QC)

Data normalization was done using the Robust Multi-array Average (RMA) from the affy (v1.82.0) package. To reduce noise, quality control checks, such as missing values and duplicate entries, were applied, along with filters for genes with low variation [13].

### 4.3. Detection of Shared Significant Genes (Exploratory Analysis)

Up- and down-differentially expressed genes between biofilm-producer and planktonic bacterial strains were identified using the limma R package (v3.60.6). Genes with an adjusted *p*-value < 0.05 and an absolute log2 fold change >1 were considered significantly differentially expressed. The overlapping DEGs from the four datasets were analyzed using a Venn diagram in R version 4.4.0. [13].

### 4.4. Integrative Analysis Using Complementary Approaches

Herein, we used an integrative analysis to capture candidate genes associated with *P. aeruginosa* biofilm formers. According to this concept, and to utilize a complementary strategy, we analyzed the same batch-corrected combined dataset using both WGCNA and limma in parallel.

#### 4.4.1. Weighted Gene Co-Expression Network Analysis (WGCNA)

We employed the WGCNA pipeline, as implemented in the R package WGCNA (v 1.73), with modifications [15]. To pool these heterogeneous datasets, only genes common to all datasets were retained, and batch effects were removed using ComBat (sva package, v3.52.0) from the sva package (version 3.52.0). We initially constructed a signed weighted gene co-expression network. The adjacency matrix was created by raising the correlation matrix to the soft-thresholding power β = 13 using pickSoftThreshold. Adjacency matrices were converted to a topological overlap matrix (TOM). Using a hierarchical clustering method based on TOM dissimilarity and dynamic tree cutting (minimum module size = 30), gene modules were identified and assigned a unique color to distinguish them. Module eigengenes were calculated, and modules were correlated with the biofilm vs. planktonic trait to assess biological relevance, using a |correlation| evaluation [15].

#### 4.4.2. Differentially Expressed Gene (DEG) Analysis

After batch effect removal, the combined dataset was analyzed [13]. Significant DEGs (biofilm-producing vs. planktonic) bacterial strains were detected using the limma R package (v3.60.6). Genes with an adjusted *p*-value < 0.05 and an absolute log2 fold change >1 were considered significantly differentially expressed. A heatmap and volcano plot were constructed in the R software (version 4.4.0) to represent the results.

### 4.5. Functional Enrichment Analysis

Candidate genes of *P. aeruginosa* were analyzed for biological functions and pathways using GO and pathway enrichment analyses. Using the enrichment features in the STRING database (https://string-db.org/), a list of candidate gene symbols was uploaded. The enrichment types were based on GO (BP, MF, and CC) terms, and KEGG pathways. In all STRING analyses, the false discovery rate (FDR) was used to assess statistical significance. Terms with an FDR < 0.05 were considered significant [54,55]. Our results were visualized in R software, using the ggplot2 package.

### 4.6. Protein–Protein Interaction (PPI) Analysis and Hub Genes Detection

The PPI networks for the significant candidate genes were constructed using the STRING database (cutoff score ≥ 0.7; high confidence) (https://string-db.org/) and analyzed in Cytoscape (v 3.10.2). Hub genes were identified in Cytoscape using the cytoHubba plugin (v0.1) [13]. Multiple algorithms (Degree, Closeness, MNC, and EPC) were used to identify the top significant hub genes in the network.

### 4.7. Multi-Domain Annotation and Data Mining of Shared Genes

The selected candidate genes were annotated by using the *Pseudomonas* Genome Database (www.pseudomonas.com). For each candidate gene, we examined functional and pathway enrichment by referencing GO and KEGG databases [56,57].

### 4.8. Preliminary Computational Validation Using an External Dataset

To computationally validate our identified candidate genes, preliminary independent validation was performed. A publicly available RNA-seq dataset, GSE301018 (*P. aeruginosa* (*PAO1*)/Illumina NextSeq 500), was retrieved from the GEO database. Biofilm samples (*n* = 4) for each time point (24, 48, and 72 h) and planktonic samples (*n* = 4) were included. The normalized expression data was downloaded in order to perform differential expression analysis using the limma package in R. In our study, an external dataset analysis was used to provide preliminary computational evidence supporting our candidate genes, rather than definitive confirmation of all of them.

## 5. Conclusions, Limitations, and Future Directions

While our hypothesis-generating integrative strategy, which combines analyses of differential expression and co-expression patterns, resulted in a list of candidate genes, it still has limitations. Although our integrative strategy increases confidence in our predictions through different steps, it does not replace external validations. In conclusion, the genes identified in this study are considered candidates based on computational predictions rather than experimental/functional validation. Further validation is recommended, including large-scale in silico analyses and in vitro experimental studies. These studies are needed to confirm their potential roles in biofilm formation, persistence, and virulence in *P. aeruginosa*.

## Data Availability

Data derived from public domain resources. The data presented in this study were derived from the following resources available in the public domain: [Gene Expression Omnibus (https://www.ncbi.nlm.nih.gov/geo/, accessed on 1 October 2025 and 1 April 2026)]. The code used in this study is available from the corresponding author upon reasonable request.

## Supplementary Materials

The following supporting information can be downloaded at https://www.mdpi.com/article/10.3390/ijms27125407/s1.

## Abbreviations

The following abbreviations are used in this manuscript:

MDR	Multi-Drug Resistant
DTR	Difficult to Treat
WGCNA	Weighted Gene Co-Expression Network Analysis
DEGs	Differentially Expressed Genes
GO	Gene Ontology
BP	Biological Process
CC	Cellular Component
MF	Molecular Function
KEGG	Kyoto Encyclopedia of Genes and Genomes
PPI	Protein–Protein Interaction
QS	Quorum sensing
GEO	Gene Expression Omnibus
TOM	Topological Overlap Matrix
MNC	Maximum Neighborhood Component
FDR	False Discovery Rate
