# Supplementary material for: Integrative Systems-Level Transcriptomic Network Analysis Identifies Candidate Genes Associated with Biofilm Formation and Virulence in Pseudomonas aeruginosa
Source: Int J Mol Sci. 2026 Jun 16;27(12):5407. doi: 10.3390/ijms27125407 (PMC13300146; doi:10.3390/ijms27125407)
Supplement: Supplementary file 1 [file ijms-27-05407-s001.zip › ijms-4241076-supplementary.pdf]

# Supplementary File

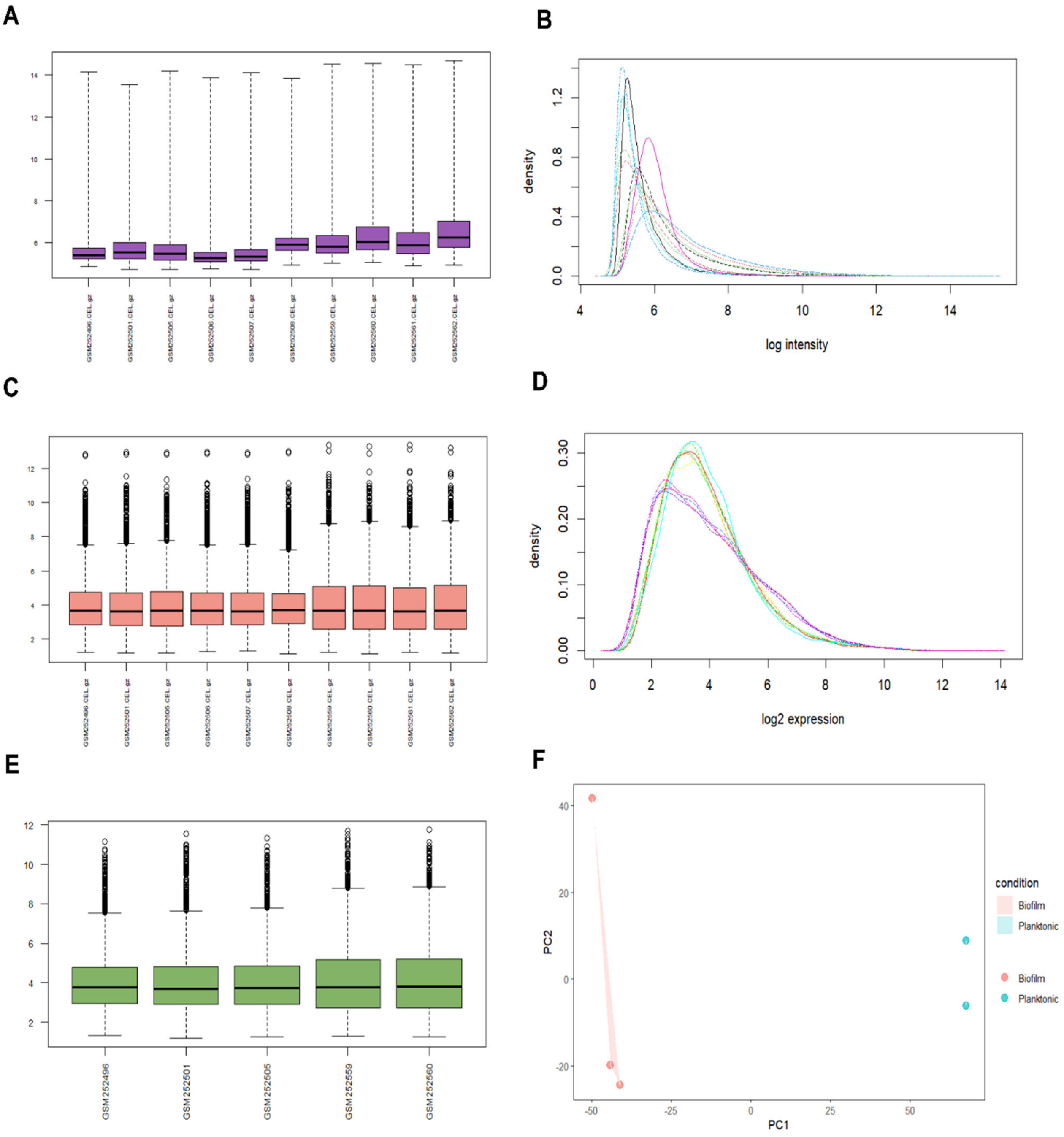


**Supplementary Material Figure S1**: Data normalization and preprocessing of dataset GSE10030. A-B) showing a boxplot and density plot of raw data. C-D) showing the boxplots and density plots of the normalized data. E) boxplots of selected subsets. F) Principal component analysis (PCA) of samples.

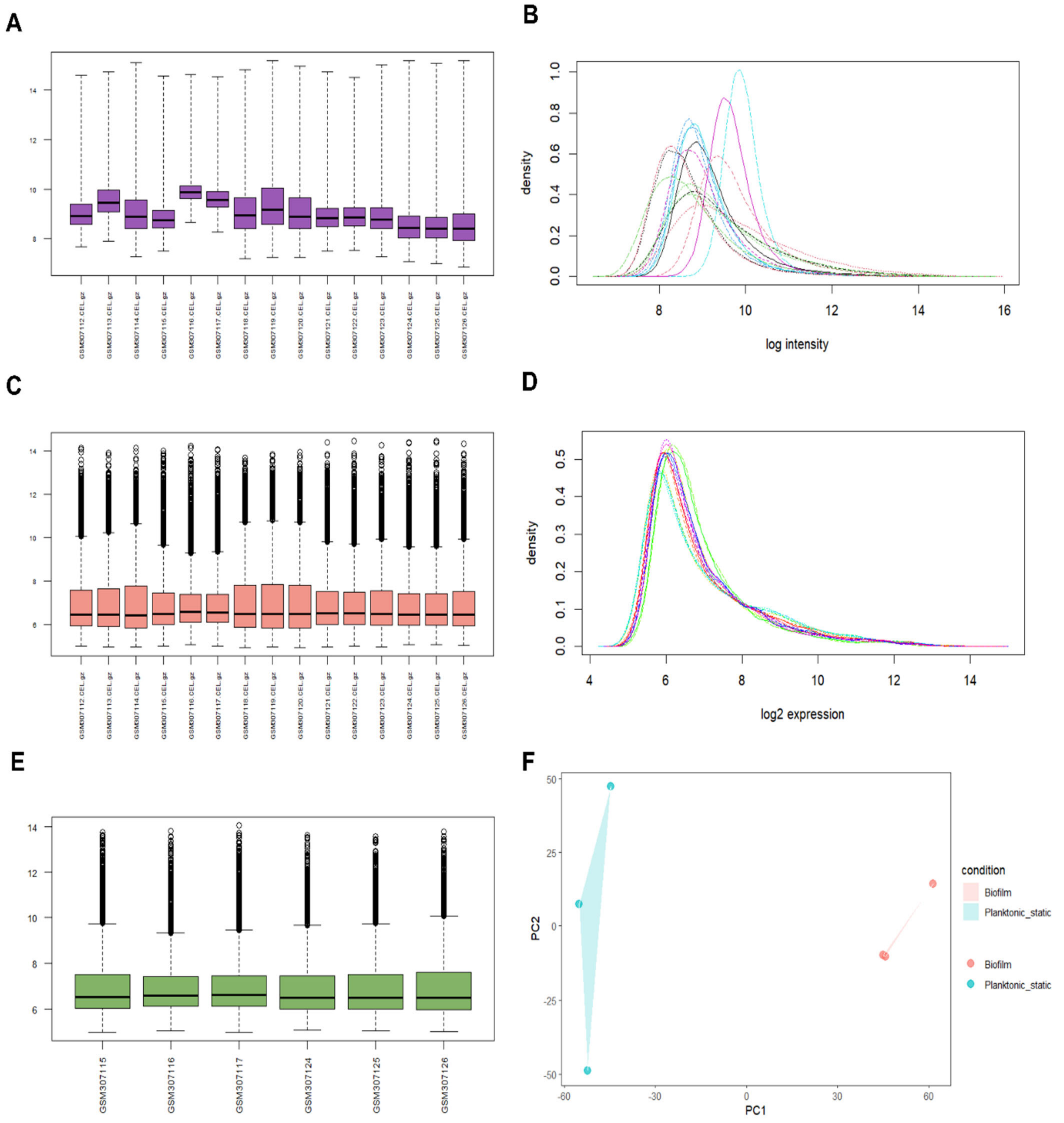


**Supplementary Material Figure S2:** Data normalization and preprocessing of dataset GSE12207. A-B) showing a boxplot and density plot of raw data. C-D) showing the boxplots and density plots of the normalized data. E) boxplots of selected subsets. F) Principal component analysis (PCA) of samples.

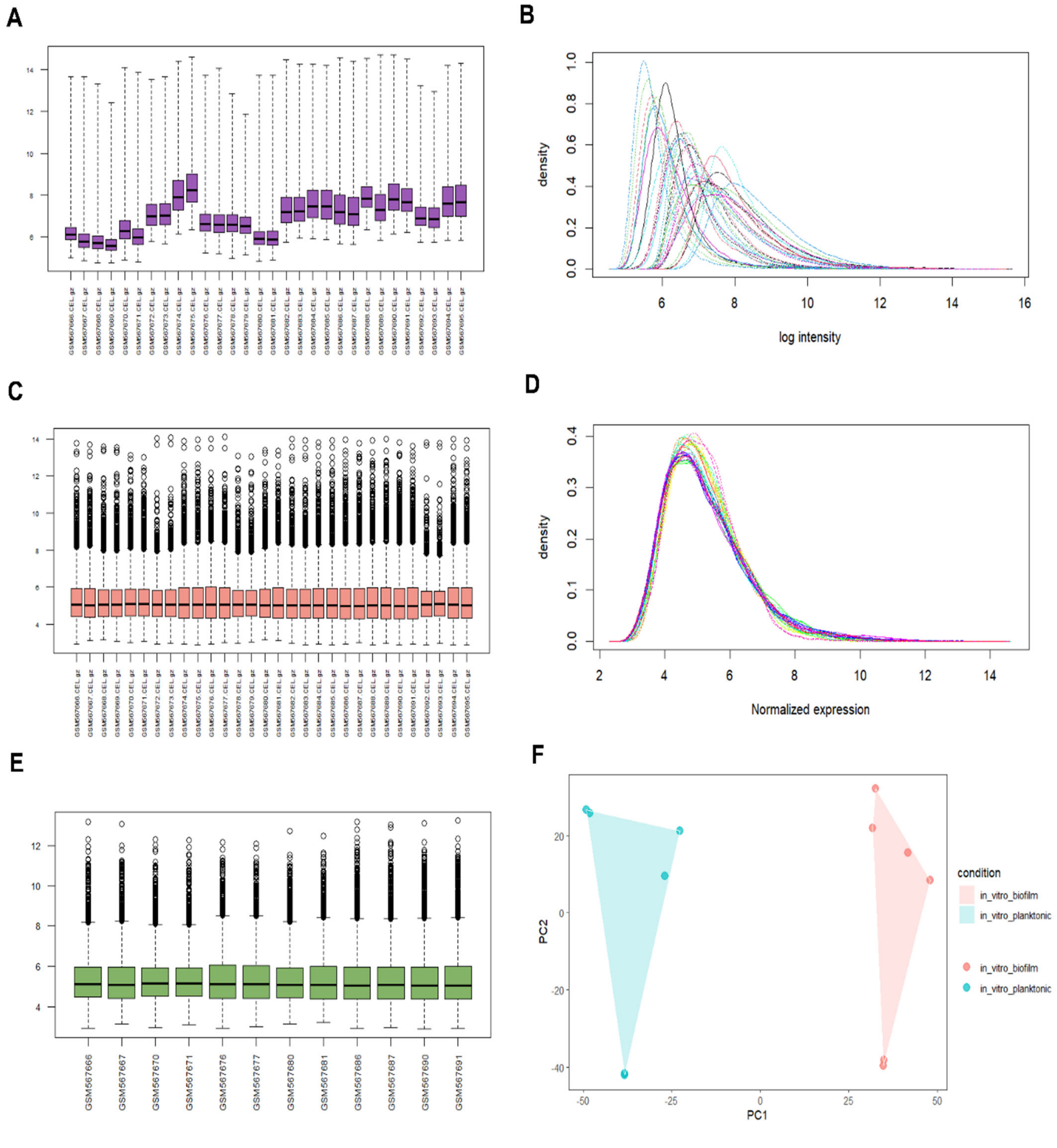


**Supplementary Material Figure S3:** Data normalization and preprocessing of dataset GSE23007. A-B) showing a boxplot and density plot of raw data. C-D) showing the boxplots and density plots of the normalized data. E) boxplots of selected subsets. F) Principal component analysis (PCA) of samples.

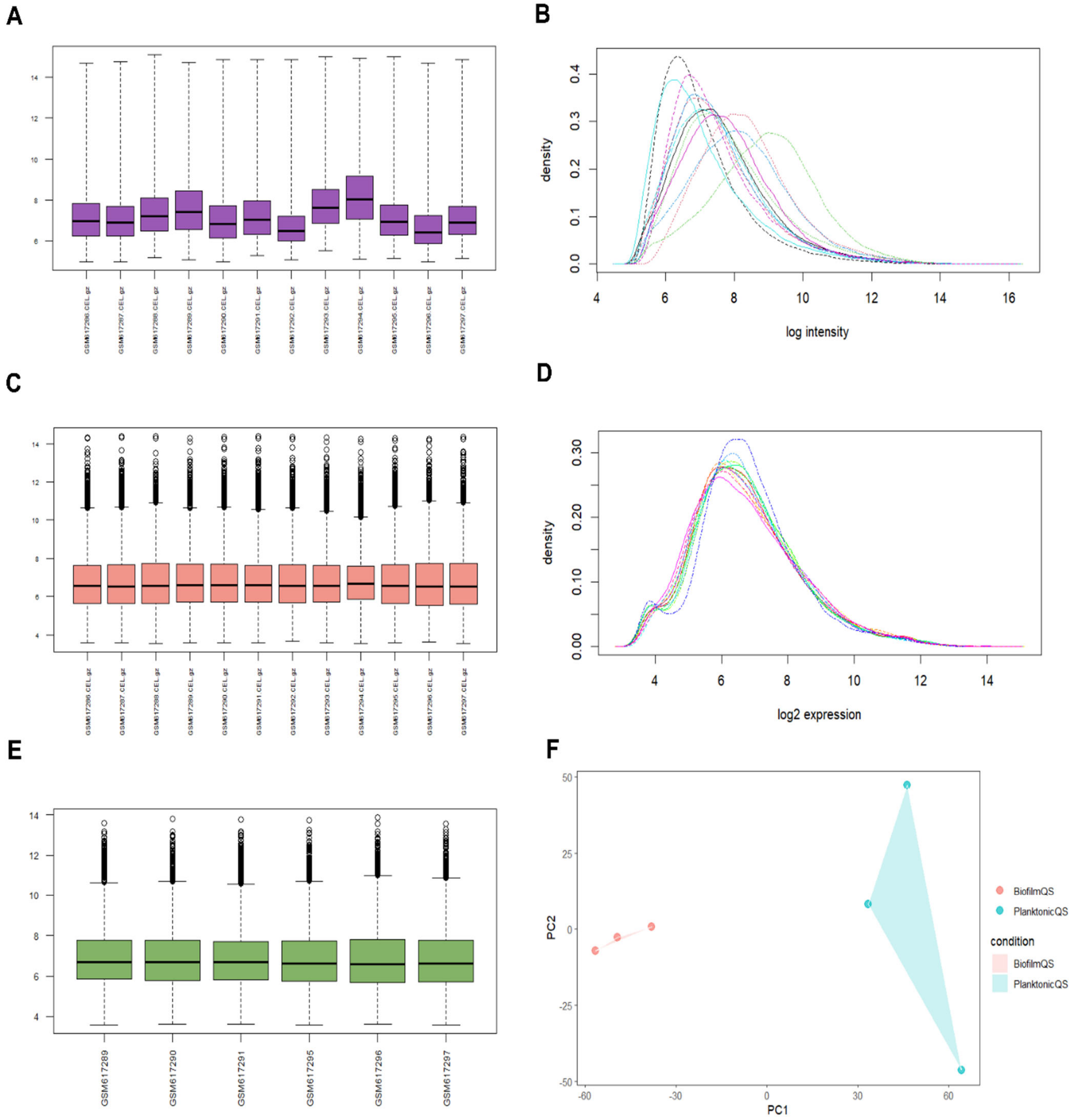


**Supplementary Material Figure S4:** Data normalization and preprocessing of dataset GSE25128. A-B) showing a boxplot and density plot of raw data. C-D) showing the boxplots and density plots of the normalized data. E) boxplots of selected subsets. F) Principal component analysis (PCA) of samples.

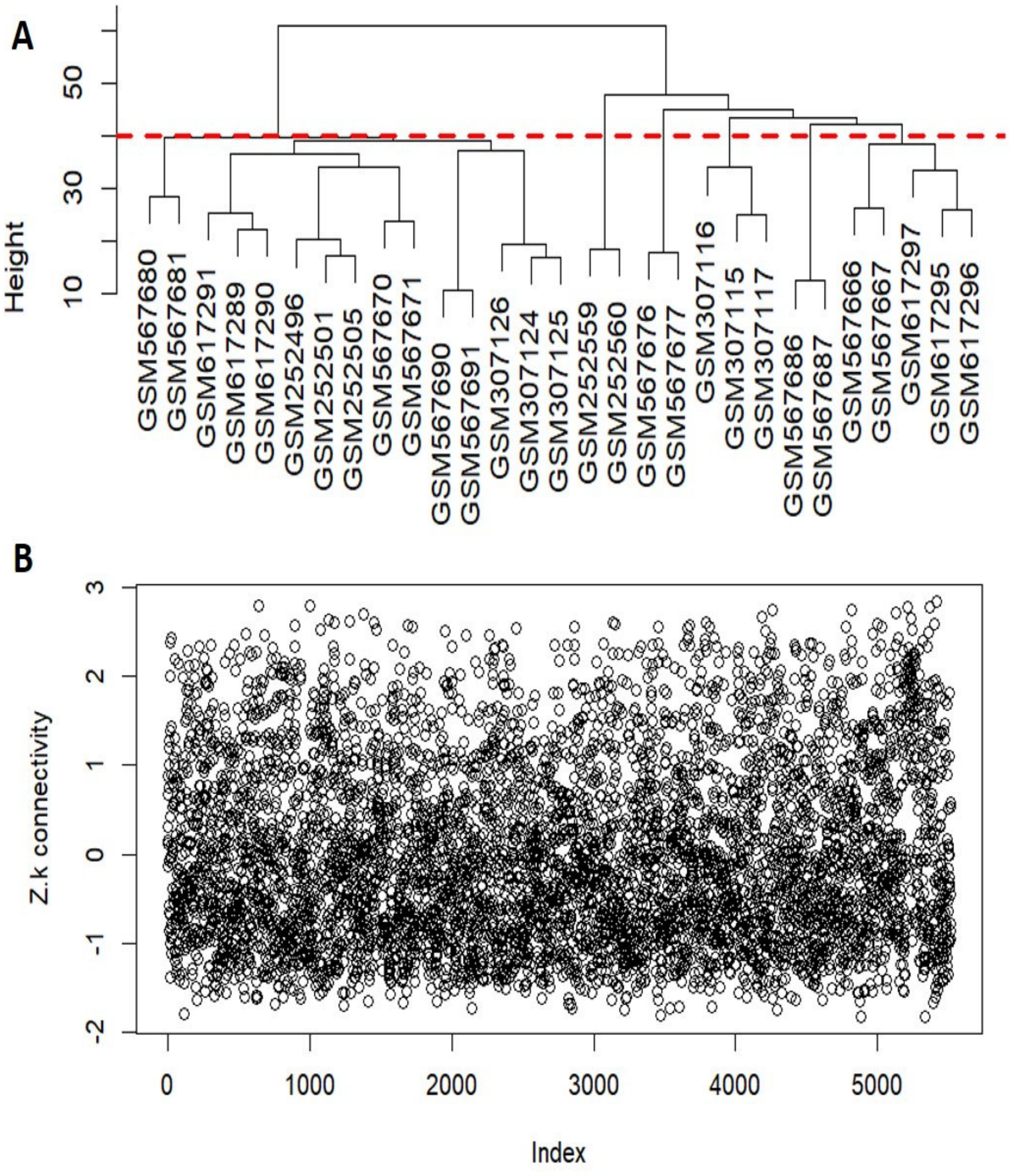


**Supplementary Material Figure S5:** Quality check and outliers detection. A) Samples clustering dendrogram. B) Samples connectivity.

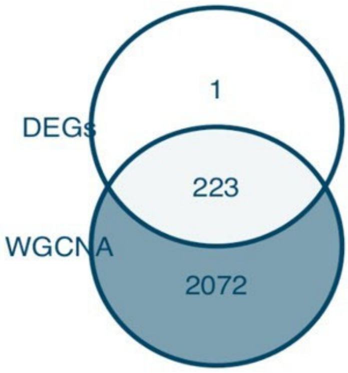


**Supplementary Material Figure S6:** Venn Diagram Showing Shared Significant Candidate Genes (n=223) Resulted from WGCNA and *Limma* Intersection. Common genes (*adk, capB, ccmH, fabZ, fis, gltP, grx, hcpC, hisG, infA, ispB, lpxA, minE, mltD, mreC, mreD, nusG, opr86, PA0578, PA0608, PA1768, PA2667, PA2840, PA2971, PA3741, PA3747, PA3806, PA3906, PA3907, PA3967, PA4461, PA4672, PA4673, PA4746, PA4757, PA5130, PA5298, PA5504, PA5568, pstB, pstS, pyrH, queA, rbfA, rho, rplI, rplS, rpmA, rpmB, rpmE, rpmF, rpmG, rpsI, rpsO, rpsT, secF, secG, speD, sspA, tpiA, typA, accB, accC, acnB, acpP, argG, aroQ1, atpA, atpB, atpC, atpD, atpE, atpF, atpG, atpH, carB, cc4, dctA, efp, eno, fabA, fabH2, fdxA, fusA1, gatC, gltA, glyA3, guaB, hcp1, hslU, infB, iscR, lpxD, lysS, mreB, murA, nusA, omlA, oprE, ostA, PA0070, PA0083, PA0084, PA1123, PA1656, PA1657, PA1658, PA1659, PA1701, PA2453, PA2760, PA2950, PA2983, PA3262, PA3331, PA3332, PA3822, PA4317, PA4429, PA4430, PA4431, PA4451, PA4558, PA4671, PA4933, PA5046, PA5139, PA5491, PA5505, PA5507, pckA, pilI, pilJ, pilY1, pnp, ppa, ppiD, pqsA, pqsB, pqsC, pqsE, prs, purB, rimM, rnpA, rplA, rplB, rplC, rplD, rplE, rplF, rplJ, rplK, rplL, rplM, rplN, rplO, rplP, rplQ, rplR, rplU, rplV, rplW, rplX, rpmC, rpmD, rpmH, rpmJ, rpoA, rpoB, rpoC, rpoD, rpsA, rpsB, rpsC, rpsD, rpsE, rpsF, rpsG, rpsH, rpsJ, rpsK, rpsL, rpsM, rpsN, rpsP, rpsQ, rpsR, rpsS, rpsU, sdhB, sdhC, sdhD, secB, secD, secE, secY, tig, trmD, tsf, tufA, vfr, ccoN2, ccoO2, ccoP2, glpD, pilH, cupA1, narG, narH, narI, nirC, nirJ, nirM, nirS, norB, norC, nosZ, PA0510, PA0513, PA0515, PA0525, phzS*)

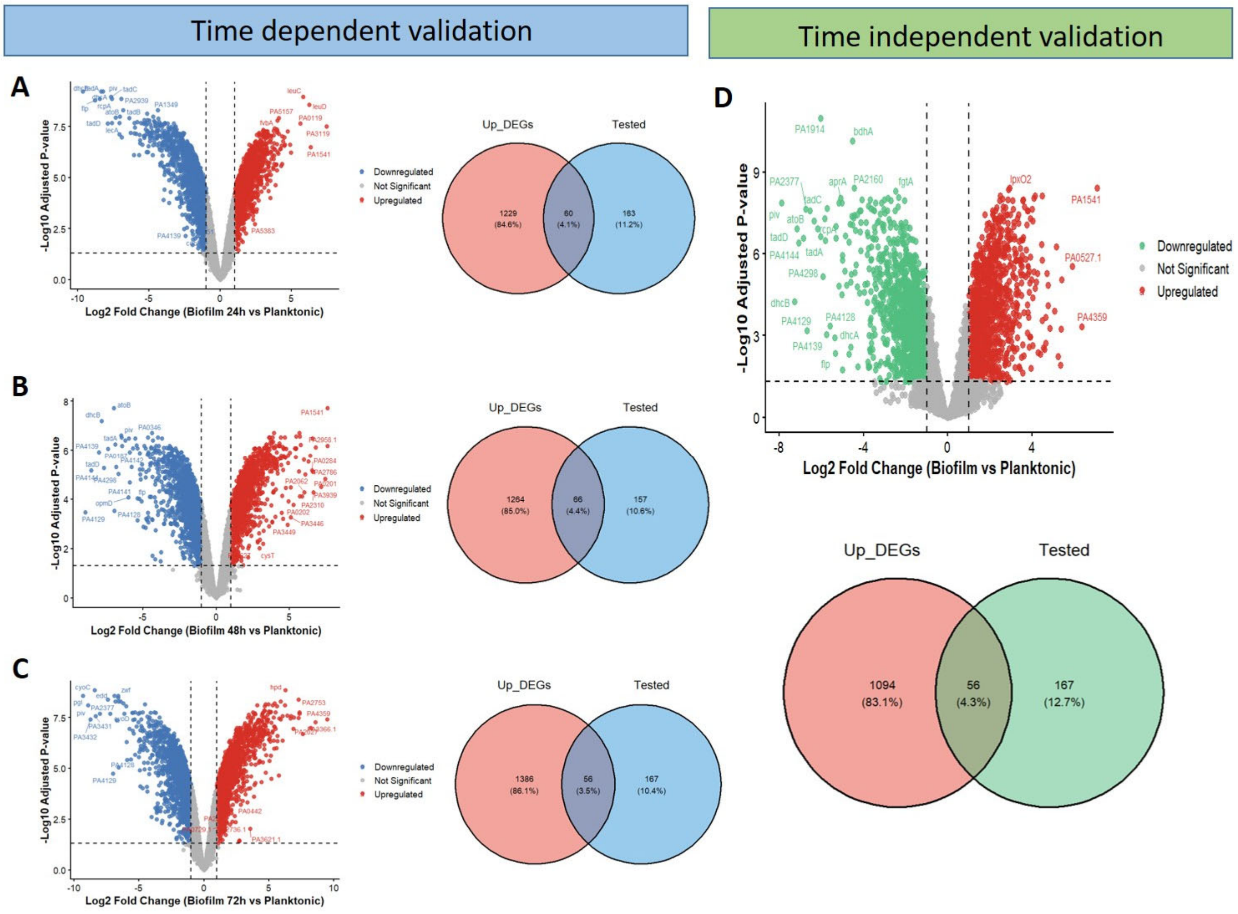


**Supplementary Material Figure S7:** External Dataset Validation (*Preliminary Validation*). Time dependent at 24 h (A), 48 h (B), and 72 h (C) biofilms. Time in-dependent (D). ***Note***: The use of the external dataset analysis was included in order to provide preliminary supportive computational evidence rather than definitive confirmation of all candidate genes. Genes confirmed under time-independent (*sdhC*, *omlA*, *secE*, *sdhD*, *PA3747*, *ccoO2*, *PA4430*, *fdxA*, *pilI*, *rpsU*, *capB*, *PA1768*, *gatC*, *ccoN2*, *ccoP2*, *PA4431*, *aroQ1*, *cupA1*, *nusG*, *PA4317*, *ispB*, *PA1123*, *queA*, *infA*, *gltA*, *nirS*, *PA4429*, *rpmF*, *pilH*, *PA2453*, *rpmH*, *fabA*, *fabZ*, *gltP*, *rpsH*, *rho*, *PA4451*, *PA5491*, *speD*, *secG*, *sspA*, *rpmE*, *mltD*, *minE*, *norB*, *prs*, *PA4757*, *PA2983*, *purB*, *PA0608*, *argG*, *rnpA*, *acpP*, *norC*, *rpsT*, *PA5139*).

# Supplementary File

**Supplementary Material Table S1. Scale-free topology model fit and connectivity metrics for selection of soft-thresholding power for weighted gene co-expression network analysis.**

| Power | SFT.R.sq | slope | truncated.R.sq | mean.k. | median.k. | max.k. |
|---|---|---|---|---|---|---|
| **1** | 0.16303866 | 6.996650 | 0.9029024 | 2787.61910 | 2788.968935 | 3009.7605 |
| **2** | 0.04183800 | 1.915569 | 0.9489240 | 1573.25493 | 1574.308740 | 1859.4482 |
| **3** | 0.05121037 | -1.297585 | 0.9405269 | 961.17684 | 955.707712 | 1305.9311 |
| **4** | 0.07177850 | -1.037761 | 0.9064203 | 623.55527 | 612.650834 | 967.6112 |
| **5** | 0.12855384 | -1.000572 | 0.8583976 | 424.03248 | 408.867299 | 744.9934 |
| **6** | 0.24334622 | -1.012277 | 0.8641951 | 299.51617 | 280.993031 | 592.4509 |
| **7** | 0.42374479 | -1.052357 | 0.9156415 | 218.29538 | 198.786207 | 484.5981 |
| **8** | 0.58845933 | -1.210924 | 0.9428214 | 163.33859 | 143.475679 | 414.5373 |
| **9** | 0.70059785 | -1.314145 | 0.9596733 | 124.98652 | 105.277464 | 359.5710 |
| **10** | 0.76281157 | -1.413837 | 0.9579697 | 97.50586 | 78.760230 | 315.5711 |
| **11** | 0.81967861 | -1.475156 | 0.9667918 | 77.35952 | 59.771247 | 280.4471 |
| **12** | 0.84215863 | -1.524558 | 0.9674618 | 62.29162 | 46.012704 | 251.1609 |
| 13 | 0.85879267 | -1.563746 | 0.9638399 | 50.82122 | 35.579567 | 226.4175 |
| **14** | 0.87870835 | -1.576822 | 0.9680910 | 41.95117 | 27.767102 | 205.2755 |
| **15** | 0.89374840 | -1.580312 | 0.9679372 | 34.99479 | 21.866277 | 187.1174 |
| **16** | 0.90187569 | -1.595191 | 0.9685352 | 29.46963 | 17.352432 | 171.3700 |
| **17** | 0.90485004 | -1.599921 | 0.9677390 | 25.03057 | 13.936701 | 157.5563 |
| **18** | 0.90574842 | -1.603685 | 0.9635740 | 21.42669 | 11.193926 | 145.3577 |
| **19** | 0.90844573 | -1.600016 | 0.9633266 | 18.47281 | 9.068056 | 134.5208 |
| **20** | 0.91580987 | -1.588564 | 0.9646279 | 16.03044 | 7.408219 | 124.8423 |

**Supplementary Material Table S2: WGCNA Modules correlation and p-values.**

| Module | Correlation | P-value |
|---|---|---|
| MEblue | 0.88786231 | 1.340292e-10 ($1.340292 \times 10^{-10}$) |
| MEyellow | 0.87501275 | 5.357851e-10 ($5.357851 \times 10^{-10}$) |
| MEred | 0.62210095 | 3.145824e-04 ($3.145824 \times 10^{-4}$) |
| MEpurple | 0.53592565 | 2.731511e-03 ($2.731511 \times 10^{-3}$) |
| MEdarkred | 0.48706131 | 7.370229e-03 ($7.370229 \times 10^{-3}$) |
| MEblack | 0.42309189 | 2.220795e-02 ($2.220795 \times 10^{-2}$) |
| MEgreen | 0.41128543 | 2.665386e-02 ($2.665386 \times 10^{-2}$) |
| MEmidnightblue | 0.26992590 | 1.567463e-01 ($1.567463 \times 10^{-1}$) |
| MEbrown | 0.24445327 | 2.012461e-01 ($2.012461 \times 10^{-1}$) |
| MEdarkturquoise | 0.23362688 | 2.225616e-01 ($2.225616 \times 10^{-1}$) |
| MElightcyan | 0.22253092 | 2.459377e-01 ($2.459377 \times 10^{-1}$) |
| MEdarkgreen | 0.03578361 | 8.537915e-01 ($8.537915 \times 10^{-1}$) |
| MEsalmon | -0.02602227 | 8.934085e-01 ($8.934085 \times 10^{-1}$) |
| MEroyalblue | -0.14066645 | 4.667226e-01 ($4.667226 \times 10^{-1}$) |
| MEtan | -0.19358787 | 3.143167e-01 ($3.143167 \times 10^{-1}$) |
| MElightgreen | -0.27361758 | 1.509348e-01 ($1.509348 \times 10^{-1}$) |
| MEgrey | -0.27714974 | 1.455209e-01 ($1.455209 \times 10^{-1}$) |
| MEdarkgrey | -0.31429263 | 9.682409e-02 ($9.682409 \times 10^{-2}$) |
| MElightyellow | -0.38110272 | 4.137699e-02 ($4.137699 \times 10^{-2}$) |
| MEmagenta | -0.50335182 | 5.378406e-03 ($5.378406 \times 10^{-3}$) |
| MEgrey60 | -0.58632080 | 8.302293e-04 ($8.302293 \times 10^{-4}$) |
| MEcyan | -0.59141822 | 7.280169e-04 ($7.280169 \times 10^{-4}$) |
| MEgreenyellow | -0.60353043 | 5.280852e-04 ($5.280852 \times 10^{-4}$) |
| MEpink | -0.61184890 | 4.203983e-04 ($4.203983 \times 10^{-4}$) |
| MEturquoise | -0.95473102 | 9.636020e-16 ($9.636020 \times 10^{-16}$) |

**Supplementary Material Table S3: Annotations and Enrichment analysis of Candidate Genes retrieved from *Pseudomonas Genome Database* (www.pseudomonas.com ).**

| Genes Name (Synonym) | Product Name | KEGG | GO Terms | | |
|---|---|---|---|---|---|
| | | | Biological Process | Molecular Function | Cellular Component |
| ***hcpC*** | secreted protein *Hcp* | -Biofilm formation - *Pseudomonas aeruginosa*<br>-Bacterial secretion system | - | - | - |
| ***PA0083 (tssB1)*** | *TssB1* | -Biofilm formation - *Pseudomonas aeruginosa* | - | - | - |
| ***PA0084 (tssC1)*** | *TssC1* | -Biofilm formation - *Pseudomonas aeruginosa* | - | - | - |
| ***hcp1*** | *Hcp1* | -Biofilm formation - *Pseudomonas aeruginosa*<br>-Bacterial secretion system | - | - | - |
| ***pilH*** | twitching motility protein *PilH* | -Two-component system<br>-Biofilm formation - *Pseudomonas aeruginosa* | phosphorelay signal transduction system | - | - |
| ***pilI*** | twitching motility protein *PilI* | -Two-component system<br>-Biofilm formation - *Pseudomonas aeruginosa* | -Chemotaxis<br>-signal transduction | - | - |
| ***pilJ*** | twitching motility protein *PilJ* | -Two-component system | -Chemotaxis<br>-signal transduction | -transmembrane signaling receptor activity | -Membrane |

| | | | | | |
|---|---|---|---|---|---|
| | | -Biofilm formation - *Pseudomonas aeruginosa* | | | |
| ***Vfr*** | transcriptional regulator *Vfr* | -Two-component system<br>-Quorum sensing<br>-Biofilm formation - *Pseudomonas aeruginosa* | -positive regulation of multi-organism process<br>-pathogenesis<br>-regulation of transcription, DNA-templated | -DNA binding | - |
| ***pqsA*** | *PqsA* | -Quorum sensing<br>-Biofilm formation - *Pseudomonas aeruginosa* | -obsolete cofactor biosynthetic process<br>-secondary metabolite biosynthetic process | - | - |
| ***pqsB*** | *PqsB* | -Quorum sensing<br>-Biofilm formation - *Pseudomonas aeruginosa* | -secondary metabolite biosynthetic process | -transferase activity, transferring acyl groups | - |
| ***pqsC*** | *PqsC* | -Quorum sensing<br>-Biofilm formation - *Pseudomonas aeruginosa* | -secondary metabolite biosynthetic process<br>-fatty acid biosynthetic process | -transferase activity, transferring acyl groups<br>-3-oxoacyl-[acyl-carrier-protein] synthase activity | - |
| ***pqsE*** | Quinolone signal response protein | -Quorum sensing | -obsolete cofactor biosynthetic process<br>-secondary metabolite biosynthetic process | - | - |
| ***PA1657 (hsiB2)*** | *HsiB2* | -Biofilm formation - *Pseudomonas aeruginosa* | -protein secretion by the type VI secretion system | - | - |
| ***PA1658 (hsiC2)*** | *HsiC2* | -Biofilm formation - *Pseudomonas aeruginosa* | -protein secretion by the type VI secretion system | - | - |

All information were retrieved from the *Pseudomonas* Genome Database (www.pseudomonas.com ), using strain *Pseudomonas aeruginosa* PAO1 (Stover et al., 2000). KEGG: Kyoto Encyclopedia of Genes and Genomes. GO: Gene Ontology.

**Supplementary Material Table S4:** Summary of microarray datasets included in this study, with strategic rationale for heterogeneity filtering (*All information was retrieved from the NCBI Gene Expression Omnibus (GEO) database*).

| Dataset | Platform | Organism | Sample ID | Status | Characteristics/ Growth Conditions (*All information was retrieved from the NCBI Gene Expression Omnibus (GEO) database*) | Strategic Rationale | Integration Strategy (Heterogeneity Filter)* |
|---|---|---|---|---|---|---|---|
| GSE10030 | GPL84 [Pae_G1a] Affymetrix *Pseudomonas aeruginosa* Array | *Pseudomonas aeruginosa* | GSM252496 | Biofilm | *Pseudomonas aeruginosa* PA14. Biofilms growing on CFBE41o- human aiway cells grown 9.5 hours | Captures early attachment stage | **Biotic-Early Filter:** Filters out rapid-growth signatures. Filter stage- and condition-specific genes. |
| | | | GSM252501 | Biofilm | *Pseudomonas aeruginosa* PA14 grown as biofilm on CFBE41o- airway cells in culture 9.5 hours in MEM/0.4% arginine | | |
| | | | GSM252505 | Biofilm | *Pseudomonas aeruginosa* PA14 Biofilms growing on CFBE41o- human aiway cells grown 9.5 hours | | |
| | | | GSM252559 | Planktonic | *Pseudomonas aeruginosa* PA14 grown to mid-exponential phase in MEM/2%LB | | |
| | | | GSM252560 | Planktonic | *Pseudomonas aeruginosa* PA14 grown to mid-exponential phase in MEM/2%LB | | |
| GSE23007 | GPL84 [Pae_G1a] Affymetrix *Pseudomonas aeruginosa* Array | *Pseudomonas aeruginosa* | GSM567666 | Planktonic | Strain: PBCLOp10. Initial isolate source: burn wound isolate. Infection model isolate source: *in vitro* planktonic culture (early stationary phase) | Captures early-to-intermediate biofilm maturation stage | **Abiotic-Stress Filter:** Removes abiotic stress effect signatures. Filter stage-specific genes |
| | | | GSM567667 | Planktonic | Strain: PBCLOp10. Initial isolate source: burn wound isolate. Infection model isolate source: *in vitro* planktonic culture (early stationary phase) | | |
| | | | GSM567670 | Biofilm | Strain: PBCLOp10. Initial isolate source: burn wound isolate. Infection model isolate source: in vitro biofilm Biofilm - 24h biofilm on plastic slide and 10% LB | | |
| | | | GSM567671 | Biofilm | Strain: PBCLOp10. Initial isolate source: burn wound isolate. Infection model isolate source: *in vitro* biofilm Biofilm - 24h biofilm on plastic slide and 10% LB | | |
| | | | GSM567676 | Planktonic | Strain: PBCLOp11. Initial isolate source: burn wound isolate. Infection model isolate source: *in vitro* planktonic culture (early stationary phase) | | |
| | | | GSM567677 | Planktonic | Strain: PBCLOp11. Initial isolate source: burn wound isolate. Infection model isolate source: *in vitro* planktonic culture (early stationary phase) | | |
| | | | GSM567680 | Biofilm | Strain: PBCLOp11. Initial isolate source: burn wound isolate. Infection model isolate source: *in vitro* biofilm | | |

| | | | | | | | |
|---|---|---|---|---|---|---|---|
| | | | | | Biofilm - 24h biofilm on plastic slide and 10% LB | | |
| | | | GSM567681 | Biofilm | Strain: PBCLOp11. Initial isolate source: burn wound isolate. Infection model isolate source: in vitro biofilm Biofilm - 24h biofilm on plastic slide and 10% LB | | |
| | | | GSM567686 | Planktonic | Strain: PBCLOp17. Initial isolate source: burn wound isolate. Infection model isolate source: in vitro planktonic culture (early stationary phase) | | |
| | | | GSM567687 | Planktonic | Strain: PBCLOp17. Initial isolate source: burn wound isolate. Infection model isolate source: in vitro planktonic culture (early stationary phase) | | |
| | | | GSM567690 | Biofilm | Strain: PBCLOp17. Initial isolate source: burn wound isolate. Infection model isolate source: in vitro biofilm Biofilm - 24h biofilm on plastic slide and 10% LB | | |
| | | | GSM567691 | Biofilm | Strain: PBCLOp17. Initial isolate source: burn wound isolate. Infection model isolate source: in vitro biofilm Biofilm - 24h biofilm on plastic slide and 10% LB | | |
| GSE25128 | GPL84 [Pae_G1a] Affymetrix *Pseudomonas aeruginosa* Array | *Pseudomonas aeruginosa* | GSM617289 | Biofilm | Strain: UUPA38. Quorum sensing phenotype: QS+. Biofilms were grown for 5 days in Luria Bertani broth at 22°C as 1 m flow-through tube cultures (bore=5 mm; flow rate=0.5 ml/min) | Captures mature biofilm stage with controlled quorum sensing | **QS-Subtraction:** filter QS specific signals. Filter stage- and condition-specific genes. |
| | | | GSM617290 | Biofilm | Strain: UUPA38. Quorum sensing phenotype: QS+. Biofilms were grown for 5 days in Luria Bertani broth at 22°C as 1 m flow-through tube cultures (bore=5 mm; flow rate=0.5 ml/min) | | |
| | | | GSM617291 | Biofilm | Strain: UUPA38. Quorum sensing phenotype: QS+. Biofilms were grown for 5 days in Luria Bertani broth at 22°C as 1 m flow-through tube cultures (bore=5 mm; flow rate=0.5 ml/min) | | |
| | | | GSM617295 | Planktonic | Strain: UUPA38. Quorum sensing phenotype: QS+. Planktonic cultures were grown to stationary phase in 2 l Luria Bertani broth shaken at 220 rpm at 37°C . | | |
| | | | GSM617296 | Planktonic | Strain: UUPA38. Quorum sensing phenotype: QS+. Planktonic cultures were grown to stationary phase in 2 l Luria Bertani broth shaken at 220 rpm at 37°C . | | |
| | | | GSM617297 | Planktonic | Strain: UUPA38. Quorum sensing phenotype: QS+. Planktonic cultures were grown to stationary phase in 2 l Luria Bertani broth shaken at 220 rpm at 37°C . | | |
| GSE12207 | GPL84 [Pae_G1a] | *Pseudomonas aeruginosa* | GSM307115 | Planktonic | *P. aeruginosa* planktonic cells after 9h of growth (early stationary phase) | Captures mature | **Late-Stage Filter:** filter |

| | | | | | | | |
|---|---|---|---|---|---|---|---|
| | Affymetrix *Pseudomonas aeruginosa* Array | | GSM307116 | Planktonic | *P. aeruginosa* planktonic cells after 9h of growth (early stationary phase) | biofilm stage | late-stage-specific genes. |
| | | | GSM307117 | Planktonic | *P. aeruginosa* planktonic cells after 9h of growth (early stationary phase) | | |
| | | | GSM307124 | Biofilm | *P. aeruginosa* biofilms in a continuous flow system after 3 days of growth. Biofilms grown for 3 days in silicone tubes | | |
| | | | GSM307125 | Biofilm | *P. aeruginosa* biofilms in a continuous flow system after 3 days of growth. Biofilms grown for 3 days in silicone tubes | | |
| | | | GSM307126 | Biofilm | *P. aeruginosa* biofilms in a continuous flow system after 3 days of growth. Biofilms grown for 3 days in silicone tubes | | |

*During integration, we applied exclusion criteria to remove stage- and condition- specific gene signatures, retaining only genes consistently present across all biofilm conditions.
